# Behavioral Characterization of A53T Mice Reveals Early and Late Stage Deficits Related to Parkinson’s Disease

**DOI:** 10.1371/journal.pone.0070274

**Published:** 2013-08-01

**Authors:** Katrina L. Paumier, Stacey J. Sukoff Rizzo, Zdenek Berger, Yi Chen, Cathleen Gonzales, Edward Kaftan, Li Li, Susan Lotarski, Michael Monaghan, Wei Shen, Polina Stolyar, Dmytro Vasilyev, Margaret Zaleska, Warren D. Hirst, John Dunlop

**Affiliations:** Neuroscience Research Unit, Pfizer Global Research and Development, Cambridge, Massachusetts, United States of America; National Institutes of Health, United States of America

## Abstract

Parkinson's disease (PD) pathology is characterized by the formation of intra-neuronal inclusions called Lewy bodies, which are comprised of alpha-synuclein (α-syn). Duplication, triplication or genetic mutations in α-syn (A53T, A30P and E46K) are linked to autosomal dominant PD; thus implicating its role in the pathogenesis of PD. In both PD patients and mouse models, there is increasing evidence that neuronal dysfunction occurs before the accumulation of protein aggregates (i.e., α-syn) and neurodegeneration. Characterization of the timing and nature of symptomatic dysfunction is important for understanding the impact of α-syn on disease progression. Furthermore, this knowledge is essential for identifying pathways and molecular targets for therapeutic intervention. To this end, we examined various functional and morphological endpoints in the transgenic mouse model expressing the human A53T α-syn variant directed by the mouse prion promoter at specific ages relating to disease progression (2, 6 and 12 months of age). Our findings indicate A53T mice develop fine, sensorimotor, and synaptic deficits before the onset of age-related gross motor and cognitive dysfunction. Results from open field and rotarod tests show A53T mice develop age-dependent changes in locomotor activity and reduced anxiety-like behavior. Additionally, digigait analysis shows these mice develop an abnormal gait by 12 months of age. A53T mice also exhibit spatial memory deficits at 6 and 12 months, as demonstrated by Y-maze performance. In contrast to gross motor and cognitive changes, A53T mice display significant impairments in fine- and sensorimotor tasks such as grooming, nest building and acoustic startle as early as 1–2 months of age. These mice also show significant abnormalities in basal synaptic transmission, paired-pulse facilitation and long-term depression (LTD). Combined, these data indicate the A53T model exhibits early- and late-onset behavioral and synaptic impairments similar to PD patients and may provide useful endpoints for assessing novel therapeutic interventions for PD.

## Introduction

Parkinson's disease (PD) is a progressive neurodegenerative disorder characterized pathologically by the loss of dopamine neurons in the substantia nigra and the formation of intra-neuronal inclusions called Lewy bodies, which are mainly comprised of alpha-synuclein (α-syn) [1]. α-syn is a small 140 amino acid (aa) protein that is ubiquitously expressed in brain, but concentrated primarily in presynaptic vesicles [2]. Although the physiological function of α-syn is unknown, evidence suggests it may be involved in neuroplasticity and regulation of synaptic vesicles [3], [4]. Furthermore, previous studies have shown that duplication [5], triplication [6] or missense mutations (A53T [7], A30P [8] and E46K [9]) in the α-syn gene are linked to autosomal dominant PD; thus implicating its role in the pathogenesis of PD.

To better understand the role α-syn plays in PD and other neurodegenerative disorders, transgenic mice expressing wild-type or mutant α-syn have been generated [10], [11], [12], [13], [14], [15], [16], [17]. These models recapitulate the effects of α-syn production and aggregation, as well as changes in neuronal structure and function. Although mouse models do not fully reproduce the pathological changes seen in PD patients (i.e., cell loss, Lewy bodies, etc.), they provide an essential tool for examining the effects of potential early intervention, symptomatic, and disease-modifying therapies. Nevertheless, to appropriately test therapeutic compounds, it is important to identify the type, extent and onset of pathological deficits in these models. Previous studies show significant behavioral and synaptic impairment in mice over-expressing wild type [18], [19], [20] or mutant α-syn [13], [14], [21], [22], [23]; however, a majority of these studies evaluate behavioral function at a finite time point, usually late in the course of disease when behavioral alterations are apparent. Although these studies help to further our understanding of how over-expressed or mutant α-syn can alter normal behavior and synaptic plasticity in animal models of PD, they do not fully characterize the onset or extent of symptoms that develop as the disease progresses. Furthermore, many of the aforementioned studies employ behavioral tests that focus on only one aspect of the disease (i.e., motor impairment). Yet, in the human disease, patients present with a multitude of non-motor and sensorimotor deficits long before the onset of motor dysfunction. Therefore, it is important to thoroughly examine the extent of symptoms within α-syn animal models as phenotypic changes that occur prior to the onset of motor dysfunction may increase our understanding of the role α-syn plays in the disease process and ultimately provide useful endophenotypes and biomarkers to assess potential disease-modifying therapies.

Here we performed a systematic evaluation, from behavior to synaptic indices, of the temporal progression of neuronal dysfunction in the A53T transgenic mouse. This mouse expresses the human A53T α-syn variant (full-length, 140-aa isoform) directed by the mouse prion promoter, which leads to the formation of filamentous neuronal inclusions and accompanying neurodegeneration [14]. This model has been studied in the context of α-syn aggregation and toxicity [14], [24], [25]; however, the temporal course of phenotypic deficits is unknown and it is not clear whether symptoms progress with disease pathology. To this end, we examined various behavioral endpoints and electrophysiological measures in these mice at specific ages relating to α-syn pathology. We measured spontaneous locomotor activity and thigmotaxis in an open field, stress induced thermoregulation (SIH), gait abnormalities, rotarod performance, nesting behavior, acoustic startle response and Y-maze in homozygous and wild-type littermates at 2, 6 and 12 months. Finally, to determine whether α-syn-mediated synaptic deficits develop with pathology or are present early on, we evaluated hippocampal synaptic physiology in young (1–2 months) A53T mice, which has not been previously characterized in this model. Our findings demonstrate for the first time that these mice display significant fine motor, sensorimotor, and hippocampal synaptic abnormalities prior to the onset of cognitive and gross motor dysfunction.

## Materials and Methods

### Animals

Mice expressing the prion promoter driven human A53T mutation were obtained from the laboratory of Virginia Lee and bred to produce wild type, heterozygote and homozygous offspring as previously described [14]. Due to genetic drift, mice were re-derived and closely monitored to ensure proper expression of the α-syn transgene with successive breeding. Mice were derived in a C3H/C57BL/6J-F1 hybrid, and the established lines were maintained by backcrossing to a C57BL/6J background. All experimental results were generated from the N1 generation (1 backcross to C57BL/6J) with homozygous mice that have 64–66 copies of the transgene. For all experiments, male transgenic mice were directly compared to their wild-type littermates. Upon arrival to the animal facility, all mice were individually housed in temperature- and humidity-controlled rooms with an automatic 12/12 h light/dark cycle with food and water ad *libitum*. All experiments were performed in accordance with the specifications of both the National Institutes of Health Guide for the Care and Use of Laboratory Animals. Studies were approved under protocol #16329 by Pfizer’s Internal Animal Care and Use Committee (IACUC). Minimal pain and suffering were involved in the experiments and mice were sacrificed either by CO_2_ asphyxiation or anesthetized with isofluorine prior to cervical dislocation.

### Survival Analysis

Thirty male homozygous A53T mice (6 months of age) were individually housed and observed on a daily basis for the onset of symptoms including, but not limited to: neglect of grooming, weight loss, and reduced ambulation. Once these symptoms became present, animals were monitored more closely until the first onset of limb paralysis at which time they were sacrificed.

### Behavioral Testing

Behavioral testing was conducted on 2 or 3 separate occasions using independent cohorts of A53T mice and their wild type littermates at 2, 6 and 12 months of age. To obtain adequate numbers (n = 8–15 per group), each age group varied in age +/− two weeks (i.e., 2 months +/−2 weeks). For all experiments, mice were acclimated to the testing room before the start of each experiment for a minimum of 30 and a maximum of 60 minutes. The inter-assay interval for behavioral testing within a cohort was typically 2 days to 1 week.

#### Open-Field test

Locomotor activity was assessed in standard Versamax Chambers (40×40×40) in a testing room with standard lighting (∼250–400 lux) and background noise (∼62–65 dB). Mice were placed in individual Versamax chambers and behaviors were recorded via infrared beams over a period of 60 minutes (5 minute time bins). The analysis included distinguishing activity within an outer and inner zone of the open field to assess thigmotaxis, total distance traveled (cm), horizontal activity (beam breaks), and vertical activity/rearing behavior (beam breaks).

#### Rotarod

The Rotamex-5 (Columbus Instruments, OH) was used to examine motor impairing phenotypes (ataxia). The drum was slowly accelerated from a speed of 4 to 40 rpm over a 300 second duration. The latency to fall off the rotarod within this period was recorded by Columbus instruments rotamex software. Mice received five consecutive trials in one day. The mean latency to fall off the rotarod for independent and combined trials was recorded and used for analysis.

#### Stress-Induced hyperthermic responses

The initial body temperature (T1) was recorded via a rectal probe (°C), then a second rectal temperature (T2) is recorded 10 minutes following T1. The stress of the initial rectal temperature recording induces an endogenous increase in body temperature known as the stress-induced hyperthermic response SIH = ΔT (T2°–T1°).

#### Nest building

To evaluate the quality of nest construction, a single A53T or wild-type mouse was placed in a polycarbonate micro-isolator cage (18 cm W×19 cm L×12 cm H) pre-filled with ∼300 g of corncob bedding lining the floor with food and water available ad libitum. One cotton nestlet (Ancare, Bellmore, NY) was placed on the cage floor prior to placing the mouse into the cage during the light phase of the lighting cycle (9–10 am). Mice were tested in counterbalanced groups of mixed genotypes and ages to reduce variability in housing conditions, and were examined for the quality of nests built at 1, 3, 5 and 24 hrs. An investigator blind to genotype and age scored the quality of the nests. Scoring was based on the nestlet rating scale (0–6; where 0 = no nest and 6 = perfect nest; Fig. S3) which was established in-house [26], [27].

#### Acoustic startle response

Mice were assessed for acoustic startle responses in an SR-LAB Startle Reflex system (San Diego Instruments). Mice were habituated by 5 min of background noise and then exposed to a series of acoustic stimuli (40 ms duration) ranging from 70 to 120 dB, inclusive of 10 repetitions of 0 db (no stim) measurements, as well as 10 repetitions of each stimulus (70, 80, 90, 100, 110, and 120 dB), occurring in a pseudorandom order. The inter-trial interval between each stimulus was 15 seconds. The maximal amplitude of each motor response was determined and the average of the repetitions of each stimulus was calculated for each individual subject and used for statistical analysis of mouse genotypes.

#### Auditory Brain Stem Response (ABR)

ABR was conducted at Jackson Laboratories at Bar Harbor, ME. Wild-type and A53T mice began testing at 80 dB/SPL and intensity was decreased by 10 dB/SPL increments until an ABR response was no longer seen. If a mouse did not show a clear response at 80 dB/SPL, then data was collected at the 90 dB/SPL level. Then the mice were tested at the 5 dB/SPL increment between the non-response and the last response seen was used to determine the hearing threshold.

#### Self-grooming behavior

Grooming behaviors were assessed using behavioral spectrometer chambers (Behavioral Instruments, NJ, USA) (19×20×18 cm) equipped with a single halogen light bulb that provided lighting of the field (∼200–250 lux). Grooming and locomotor behaviors were determined by pattern recognition software that combines vibration, animal weight, and infrared beams (described in detail at www.behavioralinstruments.com). Data were collected over a 30-minute test session. Eleven different grooming behaviors were measured and quantified (Table S1).

#### Limb asymmetry/Gait analysi

The ambulatory gait of mice was quantified using the DigiGait™ Imaging System (Mouse Specifics, Inc.). A video camera mounted below a motorized transparent treadmill belt captures the ventral side of each mouse while walking. DigiGait™ software generates “digital paw prints” and dynamic gait signals, representing the temporal record of paw placement relative to the treadmill belt. Numerous postural and kinematic metrics of gait dynamics were determined by dissecting the time each limb is spent in various portions of the walking phase, including paw area, paw placement angle during stance, stride length, stepping frequency, and stance width. Mice were placed on the treadmill and rapidly accelerated to 20 cm/sec at which speed data was captured for analysis. The total time of recording was approximately one minute. Agouti/brown mice were colored with a black Sharpie marker on their bellies to improve contrast for automated analysis.

#### Y –maze spatial recognition

Two month old subjects were tested in a separate experiment, while 6 and 12 month subjects were tested concurrently. Subjects were introduced into a Y-shaped arena (25 cm arm length×6 cm arm width×18 cm height [lower 10 cm black and upper 8 cm clear Perspex material]) and the trial began when the animal exited the start arm. During trial 1 (T1), two of three arms were open for exploration (start and familiar arms) and each animal was placed in the maze for 5 minutes and then removed back into their home cage. The door blocking the third arm (novel arm) was removed and bedding was mixed to remove odor-based cues prior to trial 2 (T2). Following a 60-minute delay, subjects were reintroduced into the arena with all arms open and allowed to explore for a 3 minute T2. Memory is reflected as novel arm preference during T2. Subjects were counterbalanced across two Y-mazes, both set up with the same visual cues around the maze. Data was recorded and analyzed with Noldus software with the exception of T2 entries, which were hand-scored. Subjects were excluded if entries or duration in 1 arm during T1 was greater than 70%, entries & duration in 1 arm were less than 10% during T2, or combination of 1 entry and less than 10% duration in 1 arm during T2, indicating inadequate exploration. Data were compared to chance (50% during T1 and 33.3% during T2) for arm duration/entries using the 95% confidence interval and unpaired t-test analysis between genotypes within age.

### Immunohistochemistry

Sections (6-µm) from paraffin-embedded tissue were cut on a rotary microtome, placed in a water bath (55°C), collected on a wet 0.45-µm nitrocellulose membrane, dried and weighted down overnight at 55°C. The sections on the nitrocellulose membrane were deparaffinized with xylene and rehydrated using a descending isopropanol series, pre-wetted with TBS-T and endogenous biotin was blocked using an avidin/biotin kit. Tissue was rinsed three times and incubated in 3% hydrogen peroxide for 20 minutes to block endogenous peroxidase. Tissue was rinsed again and incubated in Rat 15G7 α-syn (Enzo Life Sciences, Farmingdale, NY; 1∶200, diluted in 0.1% OA), a synthetic peptide corresponding to aa 116–131 of the human α-syn, for one hour followed by TBST rinse and incubation with biotin donkey anti-rat secondary (1∶1000) for 30 minutes. Tissue was incubated with Vector Elite ABC kit for 30 minutes, rinsed and stained with DAB (5 minutes) then Counterstained w/Mayer’s Hematoxylin nuclear stain (20 minutes). Slides were rinsed in tap water, dehydrated and cover slipped.

### Proteinase-K Digestion

We used proteinase K (PK) digestion to determine whether the α-syn seen in nigral neurons was soluble (non-aggregated) or insoluble (aggregated; [28], [29]Neumann et al., 2004). Sagittal brain sections were mounted onto gelatin-coated slides and dried for at least 8 h at 55°C. After wetting with TBS-T (10 mM Tris–HCl, pH 7.8; 100 mM NaCl; 0.05% Tween-20), the sections were digested with 50 µg/ml PK (Invitrogen) in TBS-T (10 mM Tris–HCl, pH 7.8; 100 mM NaCl; 0.1% Tween-20) for 30–45 minutes at 55°C. The sections were fixed with 4% paraformaldehyde for 5 minutes. After several washes, the sections were processed for α-syn immunostaining using standard histology protocol.

### Hippocampal Slice Preparation

Two or nine month-old male mice (A53T homozygous or wild-type littermates) were euthanized by decapitation. Brains were removed, cooled for 1–2 minutes in ice-cold (ice-mesh) cutting solution (in mM): NaCl 119; NaHCO_3_ 26; NaH_2_PO_4_ 1.25; KCl 3; CaCl_2_ 0.5; MgCl_2_ 5; Glucose 10, bubbled with carbogen (5%CO_2_/95%O_2_). Coronal slices were cut to 400 µm in ice-cold cutting solution using Leica VT 1200S vibratome, transferred to carbogen-bubbled artificial cerebrospinal fluid (aCSF), and left to recover for 0.5–1 hour at RT = 21–23°C, transferred to submerged type recording chamber and equilibrated for about 1 hour at 30°C before recordings. aCSF contained (in mM): NaCl 119; NaHCO_3_ 26; NaH_2_PO_4_ 1.25; KCl 3; CaCl_2_ 2.5; MgSO_4_ 1.3; Ascorbate 1.3; Na-Pyruvate 3; Glucose 10, bubbled with carbogen.

### Electrophysiology

fEPSP recordings were made from CA1 region of hippocampus at 30°C. The extracellular solution was aCSF as described above. The brain slice was positioned on the nylon net of submerged holding chamber (cat# BSC-PC, Warner Instruments, Hamden, CT) modified in-house to allow fEPSP recordings. Any given experiment represents parallel recordings from 4 brain slices located in 4 independent chambers. Recording pipette resistance was 1.5–2.5 MΩ when filled with 200 mM NaCl. fEPSP were evoked by 0.2 ms square current pulses delivered by a stimulus isolator (Model 4D, Getting Instruments, San-Diego, CA) through a bipolar stimulation electrode (cat# CBARD75, FHC, Bowdoin, ME). The stimulation electrode was positioned proximal to the recording pipette in a way to obtain peak amplitude fEPSP at about 4 ms post-stimulus. Both the stimulation electrode and recording pipette were positioned in the middle third of the CA1 stratum radiatum. For measuring basal synaptic transmission, fEPSPs were evoked every 60 seconds at 40% of maximal fEPSP amplitude calculated from the input-output curve (I-O curve: 0 to 100 µA in 10 µA steps). Long term depression (LTD) was induced by a standard protocol (900 pulses at 1 Hz). fEPSPs were filtered at 2 kHz and acquired by two double-channel MultiClamp 700A amplifiers and digitized at 20 kHz using DigiData 1322A and pClamp9 software (all from Molecular Devices, Union City, CA). Data were analyzed using Clampfit10 (Molecular Devices) and Excel (Microsoft). Statistical significance of the difference between two means was evaluated by one-way ANOVA (*p<0.05; **p<0.01; ***p<0.001). Unless specified otherwise, all data are means ± SEM. For all electrophysiological recordings and analyses, the experimenter was blind to genotype.

### Western Blot

Brains from 2-, 6- and 12-month-old wild-type and A53T mice were homogenized with RIPA lysis buffer (Sigma) containing protease inhibitors (Complete Mini, EDTA-free; Roche Diagnostics, Mannheim, Germany). Half-brain samples were homogenized with a mechanical homogenizer and left on ice for 30 minutes prior to centrifugation at 14,000 g for 20 minutes at 4°C. Protein concentration was determined with a BCA protein assay kit (Pierce, Thermo Scientific, Waltham MA). Lysates containing 30 µg of proteins were separated on 15% Tris glycine gels (Bio-rad, Hercules, CA) and then transferred onto nitrocellulose membranes (Invitrogen, Carlsbad, CA). The blots were probed with the following antibodies: mouse monoclonal anti-α-syn (1∶1000, LB509; Covance), rabbit polyclonal anti-actin (1∶10,000; Sigma, St. Louis, MO) goat anti-rabbit (1∶10,000), goat-anti-mouse (1∶10,000; Novus). Protein bands were detected and quantified with the OdysseyClx infrared scanning system (Li Cor, Lincoln, Nebraska).

### Statistical Analyses

Unless otherwise indicated, GraphPad Prism® v.5 was used for statistical analyses. With the exception of the nesting data, statistical comparisons between groups were done using an independent two-tailed t-test, a one-way, or two-way analysis of variance (ANOVA) depending on the experimental design. Post-hoc pair-wise comparisons utilized the Tukey HSD or Bonferroni method. All data are represented as mean ± SEM. Differences were regarded as statistically significant at *P*<0.05. A two-step nonparametric analysis of covariance (ANCOVA) was performed on the nesting data using the SAS program v9.2 [30], [31]. The age and time covariate factors were fit to the ranked nesting scores using an ANCOVA model, followed by a Cochran-Mantel-Haenszel test on the residuals to assess the effect of group on the nesting scores.

## Results

### Alpha Synuclein Increases with Age and Onset of Disease

**Figure 1:**
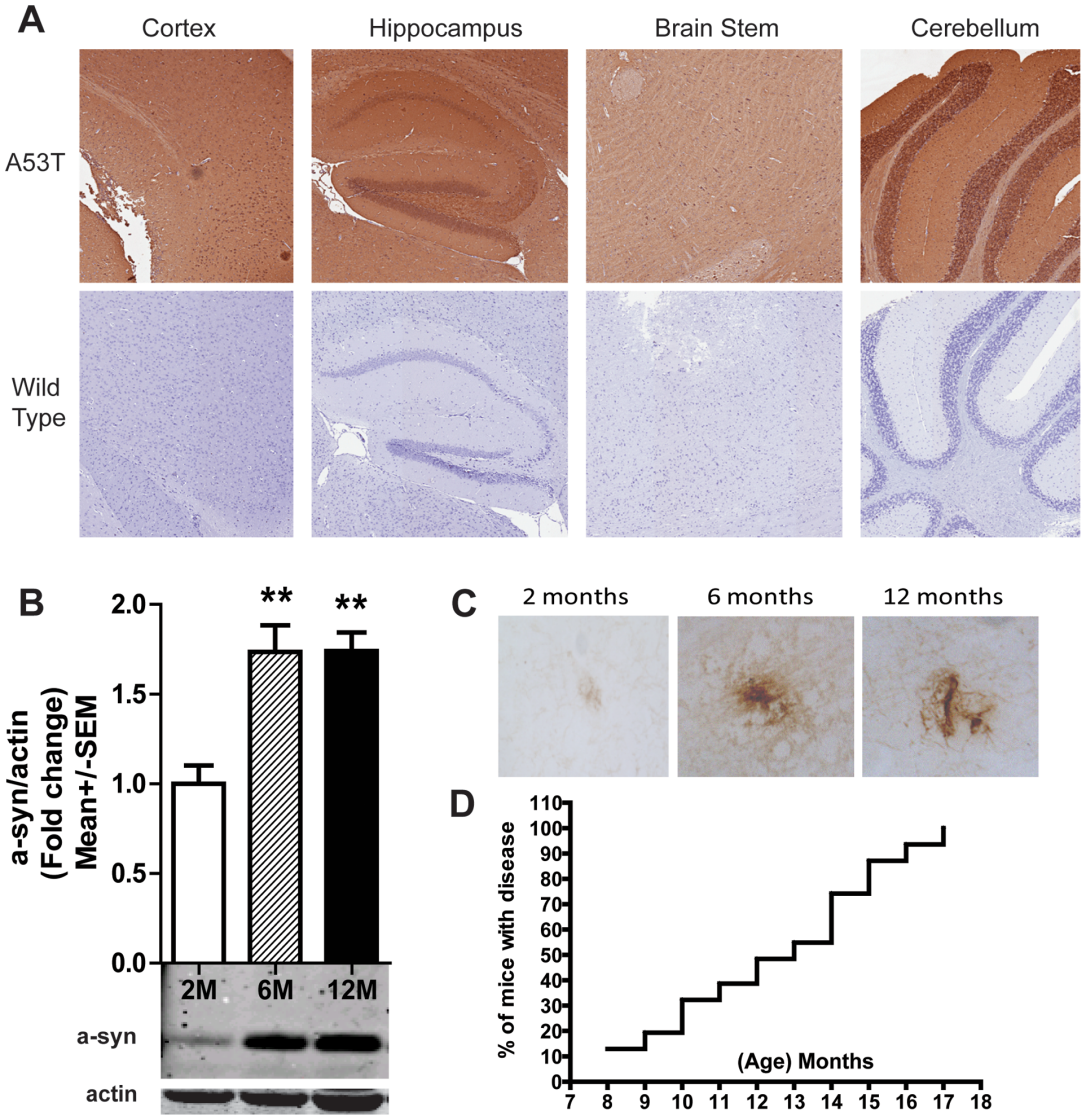
Total and aggregated mutant alpha synuclein (α-syn) increases with age and onset of disease. Human specific α-syn was expressed in neurons and neuropil of A53T homozygote (top panel), but not wild type mice (bottom panel; A). Western blot analysis indicates human α-syn levels significantly increase with age in A53T brain (n = 6 per group; B). α-syn aggregates are rare but visible at 2 months, then accumulate with age; representative photo of proteinase-K resistant aggregates in A53T brain (pons region) at 2, 6 and 12 months (C). Kaplan-Meier survival curve illustrates onset of disease, as defined by motor disability, between 8–17 months in homozygous A53T mice (n = 30; D). Data normalized to actin and plotted as Mean+/− SEM. **p<0.001.

The expression and temporal profile of α-syn was confirmed using immunohistochemistry and Western blots. Staining revealed α-syn expression in neurons and neuropil of A53T homozygote (Fig. 1A) and heterozygote (not shown), but not wild type brain (Fig. 1A). Additionally, to determine whether α-syn expression increases with age, we examined lysates from hemi-brains via Western blot analysis and results show that α-syn levels dramatically change between 2 and 6 months, with high levels also observed at 12 months (*F* (2, 13) = 13.91, *p* = 0.0006; Fig. 1B). Based on the clear differences in α-synuclein accumulation, the 2 and 6 month time points were chosen for immunohistochemical staining, with the 12 month time point added to observe further accumulation of insoluble aggregates after proteinase K digestion (Fig. 1C). Finally, we performed a survival analysis to evaluate the onset of disease in homozygous A53T mice. Mice were closely monitored beginning at 6 months of age until the onset of disability (i.e., paralysis of one limb) at which time they were euthanized. Results indicate mice develop disability between 8 and 16 months of age (Fig. 1D) as previously described [14].

### A53T and Wild Type Mice Exhibit Significant Body Weight Differences with Age

Prior to running animals in behavioral tests, the weight of each animal was collected. We found a significant effect of genotype (*F*(1,102) = 33.98, *p*<0.0001) and age (*F*(1,102) = 63.12, *p*<0.0001) on body weight. Post hoc analyses indicate the 6 (36.29+/−1.23; p<0.01) and 12 (42.26+/−0.94; p<0.001) month wild type mice were significantly heavier than the 6 (32.55+/−0.58) and 12 (34.11+/−0.47) month A53T mice (Fig. S1 F).

### A53T Mice Develop Age-related Gross Motor Alterations

**Figure 2:**
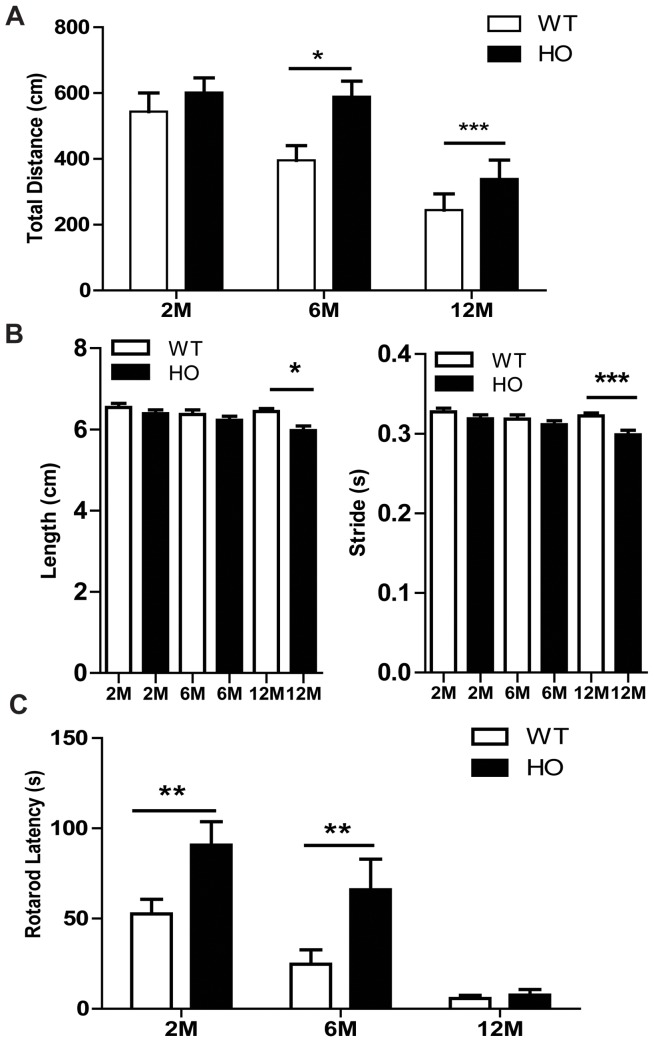
A53T homozygote mice develop gross motor abnormalities with age. In the open field, results from spontaneous locomotor testing indicate A53T mice travel significantly greater distances than wild type littermates at 6 and 12 months (A). Digigait analysis shows the stride length and frequency (steps per second) are significantly decreased in 12 month A53T mice compared to wild type littermates (t-test analysis within age group- data represent both hind limbs (B). The combined latency to fall from 5 individual trials shows A53T mice exhibit enhanced rotarod latency that declines with age (C). Graphs are representative of data collected from 2–3 independent cohorts of animals with 8–15 mice per group. Data plotted as Mean+/− SEM. Bonferonni post hoc test *p<0.05, **p<0.001; s = seconds, cm = centimeters, M = month, WT = wild type, HO = homozygous.

To evaluate the temporal onset of motor disability, we examined spontaneous locomotor activity and gait asymmetry in several cohorts of mice. Not surprisingly, A53T mice develop significant alterations in motor ability with age. These data are consistent with previous findings [13], [14], [23], [32]; however, we found the onset of symptoms does not appear to be progressive in nature, but rather develops suddenly and remains constant with age. In the spontaneous locomotor test, significant differences in both genotype (*F* (1, 66) = 101.82, *p*<0.0001) and age (*F* (2, 66) = 212.77, *p*<0.0001) were observed. At 6 and 12 months, the total distance traveled was significantly greater in homozygous mice compared to their wild type littermates (Fig. 2A). For further assessment of age-dependent locomotor alterations in homozygous mice, additional cohorts of animals (2, 6 and 12 months) were tested in the open field (Fig. S1A–C). At two months, the wild type and A53T mice travel similar distances; however, as they age, the A53T mice develop a hyperactive phenotype as illustrated by the significant increase in distance traveled in homozygous mice (Fig. S1 A). Significant increases in horizontal (Fig. S1 B) and vertical (Fig. S1 C) activity were also observed with age, providing further evidence that A53T mice develop age-related increases in activity. These mice also develop gait abnormalities with age as there was a significant decrease in hind limb stride length (*F*(3,85) = 3.07, *p* = 0.0149) and frequency (strides per second) (*F*(3,85) = 3.65, *p* = 0.0158) for 12 month old A53T mice compared to wild type littermates (Fig. 2B). Data from both the right and left hind limbs were combined for analyses; however, results were significant on both sides before data were combined (Fig. S1 D).

### A53T Mice Develop Balance/coordination Deficits with Age

Balance and coordination often become difficult for PD patients; therefore, the rotarod test was used to evaluate balance and coordination in A53T and WT mice. This test revealed a significant effect of age (*F* (2, 42) = 51.55, *p*<0.0001) and genotype (*F* (1, 42) = 13.18, *p*<0.0001), which suggests balance/coordination is altered in both wild type and homozygous mice as they age (Fig. 2C). Post hoc analyses show a significant increase in the latency to fall at 2 and 6 months in A53T mice compared to wild type littermates (p<0.0001); however, both wild type and A53T mice show an abrupt decrease in the latency to fall at 12 months (Fig. S1 C).

### A53T Mice Exhibit an Anxiolytic Phenotype with Age

**Figure 3:**
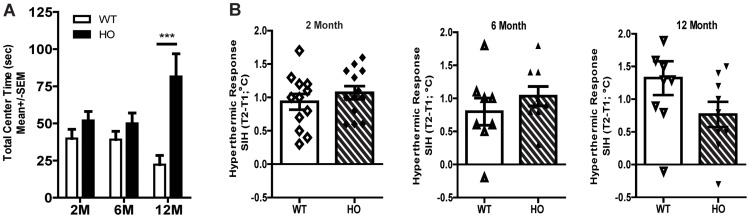
A53T mice develop a reduced anxiety–like phenotype with age. Mice were allowed to freely explore a brightly lit open field for 60 minutes. Graph shows the total time spent in the center of the field from 2-, 6- and 12-month wild type and A53T mice (A). A53T mice spend significantly more time in the center of the open field at 12 months of age compared to wild type littermates, suggesting a reduced anxiety–like phenotype. The initial body temperature (T1) was recorded via a rectal probe (°C), then a second rectal temperature (T2) is recorded 10 minutes following T1. The stress of the initial rectal temperature recording induces an endogenous increase in body temperature known as the stress-induced hyperthermic response SIH = ΔT (T2°–T1°). Graphs show the hyperthermic response from 2-, 6- and 12-month wild type and A53T mice (B). Although not statistically significant (p = 0.177), there was a trend for an attenuated Stress-Induced hyperthermic (SIH) response relative to wild type control in 12 month old mice, which may be indicative of an anxiolytic-like phenotype. Graphs are representative of data collected from 2–3 independent cohorts of animals with 8–15 mice per group. Bonferonni post hoc test ***p<0.0001; WT = Wild type, HO = Homozygous, M = Month, sec = seconds, T1 = Test 1, T2 = Test 2, C = Celsius.

As anxiety is an issue for many PD patients, we sought to determine whether A53T mice develop an anxious phenotype relative to disease progression. We utilized the open field and stress-induced hyperthermic (SIH) tests to assess anxiety in wild type and A53T mice. Results indicate A53T mice develop an anxiolytic-like phenotype with age. Evaluation of the open field data show homozygous A53T mice, but not wild type littermates, display an age-related, significant increase in center time (*F* (1, 66) = 19.41, *p*<0.0001; Fig. 3A). The center time measurement is often used as a ‘soft measure of anxiety [20],[33],[34];’ therefore, these findings suggest A53T mice become less anxious with age. Next, we examined the stress induced hyperthermic (SIH) response in these mice. Previous evidence shows that animals exposed to various stressors such as heat, noise, restraint, pain, etc. respond with an increase in core body temperature [35]. Although not significant (p = 0.107), results show that 12 month old A53T mice had an attenuated SIH response relative to wild type mice, suggesting these mice do not respond to stressors the same as their wild type counterparts, which is further evidence that these mice may exhibit an anxiolytic-like phenotype as they age (Fig. 3B).

### A53T Mice Develop Spatial Memory Deficits with Age

**Figure 4:**
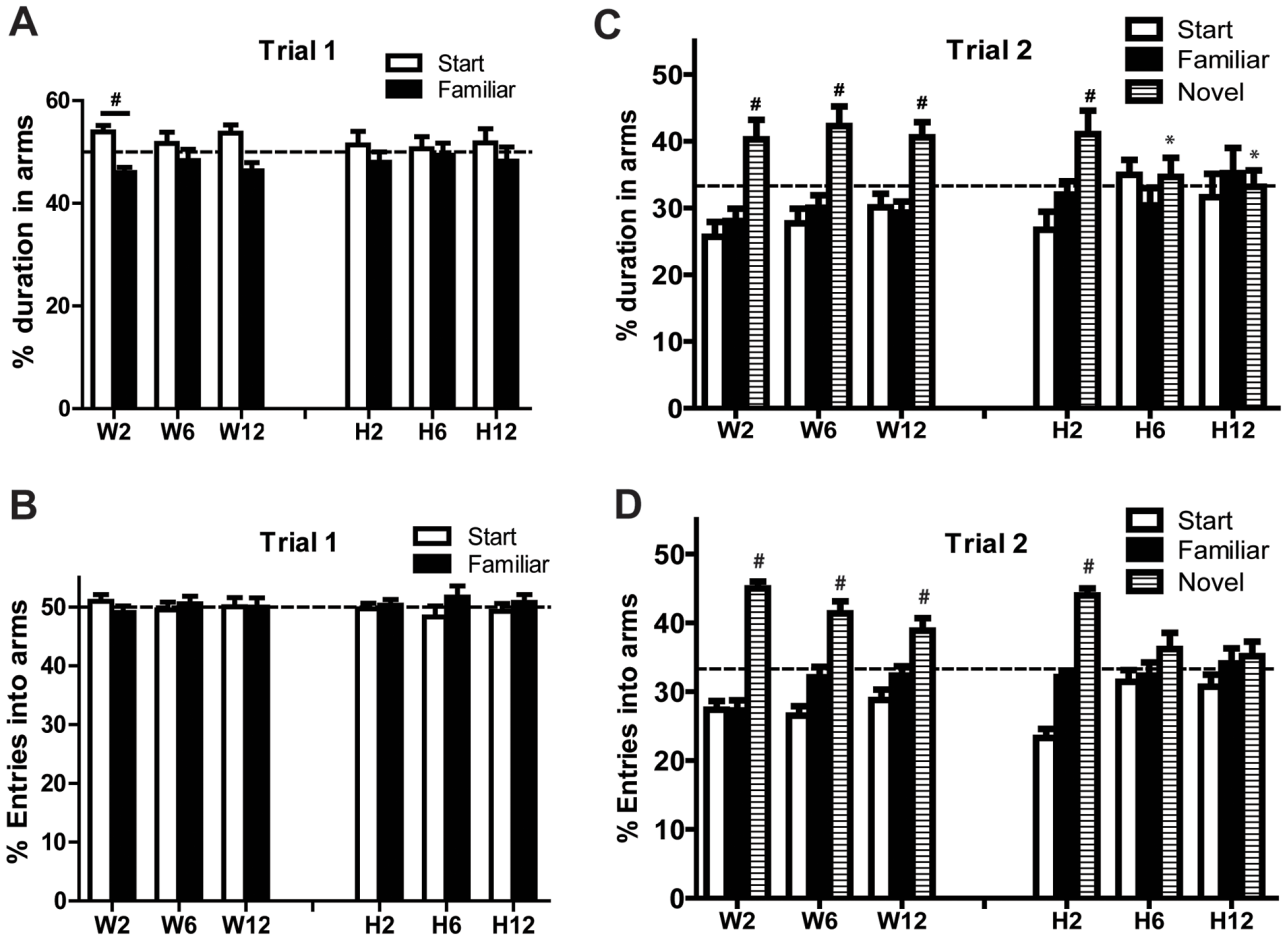
A53T mice exhibit age-related spatial memory deficits. Mice were allowed to explore two arms (start & familiar) of the Y-maze for five minutes (Trial 1). During this acclimation period, both wild type and A53T mice performed similarly when exploring (A) and entering (B) arms. The percent duration in each arm for 2 month old wild type mice was different than chance (#, 95% confidence interval; dotted line = 50%); however the overall entries and total time spent in arms were similar for both genotypes (data not shown). After a one-hour inter-trial interval, wild type mice of all ages and 2-month-old A53T mice spent significantly more time exploring (C) and entering (D) the novel arm of the Y-maze compared to the start or familiar arms. Conversely, 6 and 12 month old A53T mice did not show a preference for the novel arm, suggesting a disruption in spatial memory. There were no alterations in locomotor indices across genotype or age (not shown). Data plotted as Mean +/−SEM. To look for equal exploration of arms, data were compared to chance (depicted by dotted line; T1 = 50%, T2 = 33.3%) using the 95% confidence interval (^#^p<0.05). Unpaired t-test analysis was utilized for assessing differences between genotypes within age (*p<0.05). n = 11–15 per group; W = wild type, H = homozygote.

To assess whether A53T mice develop age-related cognitive deficits, we utilized the Y-maze two-trial recognition memory test. All mice were allowed to explore the start and familiar arms of the Y-maze for a total of 5 minutes (Trial 1: T1). Then, mice were removed and placed in their home cage. After one hour, mice were returned to the Y-maze where they were presented with a novel arm, in addition to the start and familiar arms for three minutes (Trial 2: T2). Both wild type and A53T mice performed similarly when exploring (Fig. 4A) and entering (Fig. 4B) arms during T1. The percent duration in each arm for 2 month old wild type mice was slightly different than chance (50%; 95% confidence interval); however the overall entry and total time spent in arms was similar for both genotypes (Fig. S2 E & G). Results from T2 show wild-type mice spent a greater percent of time exploring (Fig. 4C) and entering (Fig. 4D) the novel arm at 2, 6 and 12 months of age, suggesting intact spatial memory. Two month A53T mice performed similar to wild type mice (Fig. 4C, D), indicating intact memory. Conversely, the percent of time spent (ie., duration) in the novel arm was significantly less in 6 [t(22) = 2.644; p = 0.014] and 12 [t(22) = 2.222; p = 0.037] month A53T mice compared to wild type littermates of the same age, indicating A53T mice failed to recognize the novel arm during T2. This suggests a spatial memory deficit develops with age in these mice. No significant differences were found in total distance traveled, velocity, total arm entries, or total duration in all arms (Fig. S2).

### Grooming Behavior is Decreased in A53T Mice

**Figure 5:**
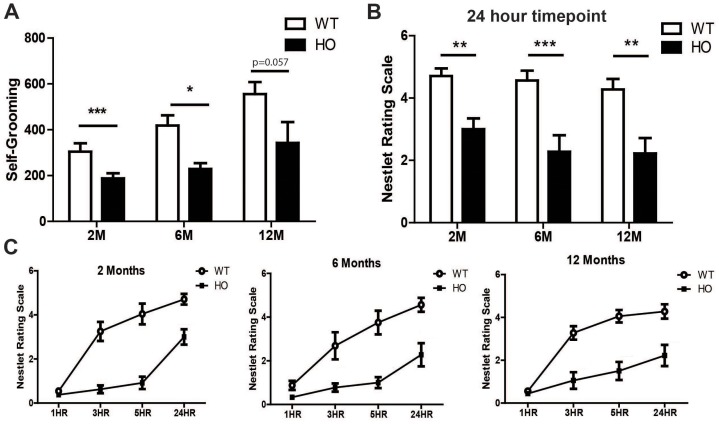
A53T mice exhibit deficits in grooming and nest building at an early age. Sensorimotor skills were assessed by the ability of mice to groom themselves and the speed and quality of nest building. Results from a combination of 11 different grooming behaviors show A53T mice groom significantly less than their wild type littermates beginning at 2 months of age (A). The nest building ability (rated on a scale of 0–6) of A53T mice is significantly impaired at the 24-hour time point in 2, 6 and 12 month mice (B). Although A53T mice build nests, they are less proficient than their wild type littermates as illustrated by the nesting time course (1, 3, 5 and 24 hours) for each age group. Age-adjusted tests by time show significant effects at 1 HR (Bonferroni adjusted p<0.01; 6 month group), as well as hours 3, 5, and 24 (Bonferroni adjusted p<0.0001; all age groups) (C). Graphs are representative of data collected from 2–3 independent cohorts of animals with 8–15 mice per group. Data plotted as Mean+/− SEM. Bonferonni post hoc test **p<0.01; ***p<0.0001; HR = Hour, M = Month, WT-Wild type, HO = Homozygous.

Grooming behavior was assessed using behavioral spectrometer chambers. Pattern recognition software uses vibrational energy, the weight of the animal, and infrared beams to analyze eleven individual grooming behaviors (listed in Table S1). Data from all behaviors were combined to produce an average “score” that was used in the analysis. A53T mice showed significant deficits in overall grooming behavior compared to wild type littermates (*F* (1, 46) = 20.29, *p*<0.0001; Fig. 5A). Additionally, there was an effect of age as both homozygous and wild type mice displayed a significant increase in grooming with age (*F* (2, 46) = 9.92, *p* = 0.0003). Combined, these data indicate that grooming behavior increases as mice age; however, A53T mice groom significantly less than their wild type littermates at all ages.

### Nesting Behavior is Altered in A53T Mice

Nest building, exhibited by both male and female mice, is a goal-directed behavior that involves species-typical sensorimotor actions important to the survival of the animal. These behaviors are dependent upon motivation, physical ability and orofacial/forelimb movements; therefore we assessed the ability of both homozygous and wild type mice to build nests by assigning a score (0–6) based on the quality of nests built after 1, 3, 5 and 24 hours (Fig. S3). Results from the 24 hour time point show a significant deficit (CMH statistic = 21.99, p<0.0001; Fig. 5B) in nest building for A53T mice at any age when compared to wild type littermates. Although A53T mice build nests, they are less proficient than their wild type littermates as illustrated by the nesting time course (1, 3, 5 and 24 hours) for each age group (Fig. 5C).

### Acoustic Startle Response is Impaired in A53T Mice

**Figure 6:**
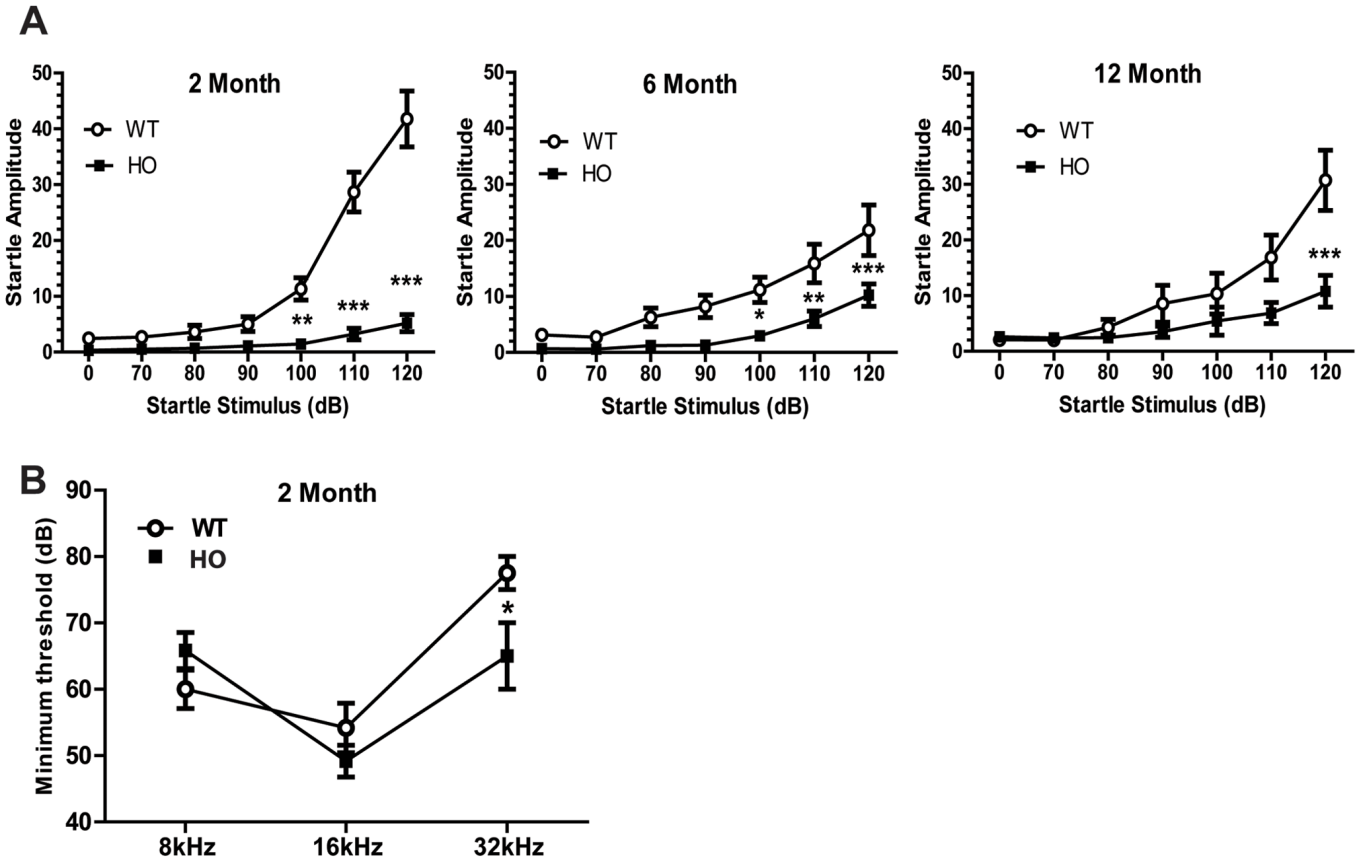
A53T mice elicit a reduced startle response at 2 months of age. The startle reflex response is significantly reduced in homozygous A53T mice relative to wild type controls at 2, 6 and 12 months (A). Results from the auditory brainstem response test suggest the reduction in startle is not due to hearing loss as there is no difference between wild type and A53T mice in hearing when exposed to frequencies of 8 and 16 kHz. In fact, A53T mice actually hear at a lower threshold when exposed to the 32 kHz frequency (N = 6 per group; 2 months of age) (B). Unless otherwise noted, graphs are representative of data collected from 2–3 independent cohorts of animals with 8–15 mice per group. Data plotted as Mean+/− SEM. Bonferonni post hoc test *p<0.05; **p<0.001; ***p<0.0001; kHz = kilohertz, dB = decibel, WT = Wild type, HO = Homozygous.

Previous studies have shown that aged A53T homozygous mice exhibit an impaired acoustic startle response prior to disease onset (∼6 months) [36], [37]; however, the temporal onset of this impairment is not known. Therefore, we evaluated 2, 6 and 12 month old mice to determine whether this reflex is impacted at a young age. Additionally, the aforementioned studies allude to the possibility that this deficit may be due to a hearing impairment. Accordingly, we conducted an auditory brainstem response test in young mice (2 months) to determine whether startle impairment is due to a sensorimotor or hearing deficit. Results from the startle response studies show a significant effect of genotype at 2 (*F* (1, 144) = 120.64, *p*<0.0001), 6 (*F* (1, 126) = 22.47, *p*<0.0001) and 12 (*F* (1, 150) = 5.31, *p* = 0.029) months of age (Fig. 6A). Bonferroni post hoc tests show a significant reduction in startle response at the 100, 110 and 120 dB for 2 and 6 month old A53T mice and a significant reduction only at the 120 dB for 12 month old A53T mice. It is notable that with age, the wild type mice display a modest decrease in startle response (Fig. S4 A), while A53T mice display an increase (Fig. S4 B). Furthermore, results from the auditory brainstem response test reveal significant differences between genotype (*F* (2, 30) = 2.04, *p*<0.0001; Fig. 6B). However, A53T mice do not exhibit a loss of hearing, in fact, they hear at a lower threshold than their wild type littermates. Taken together, these data indicate A53T mice have equivalent hearing ability and exhibit sensorimotor deficits long before the onset of motor disability or disease pathology.

### A53T Mice Exhibit Alterations in Basal Synaptic Transmission and Synaptic Plasticity

**Figure 7:**
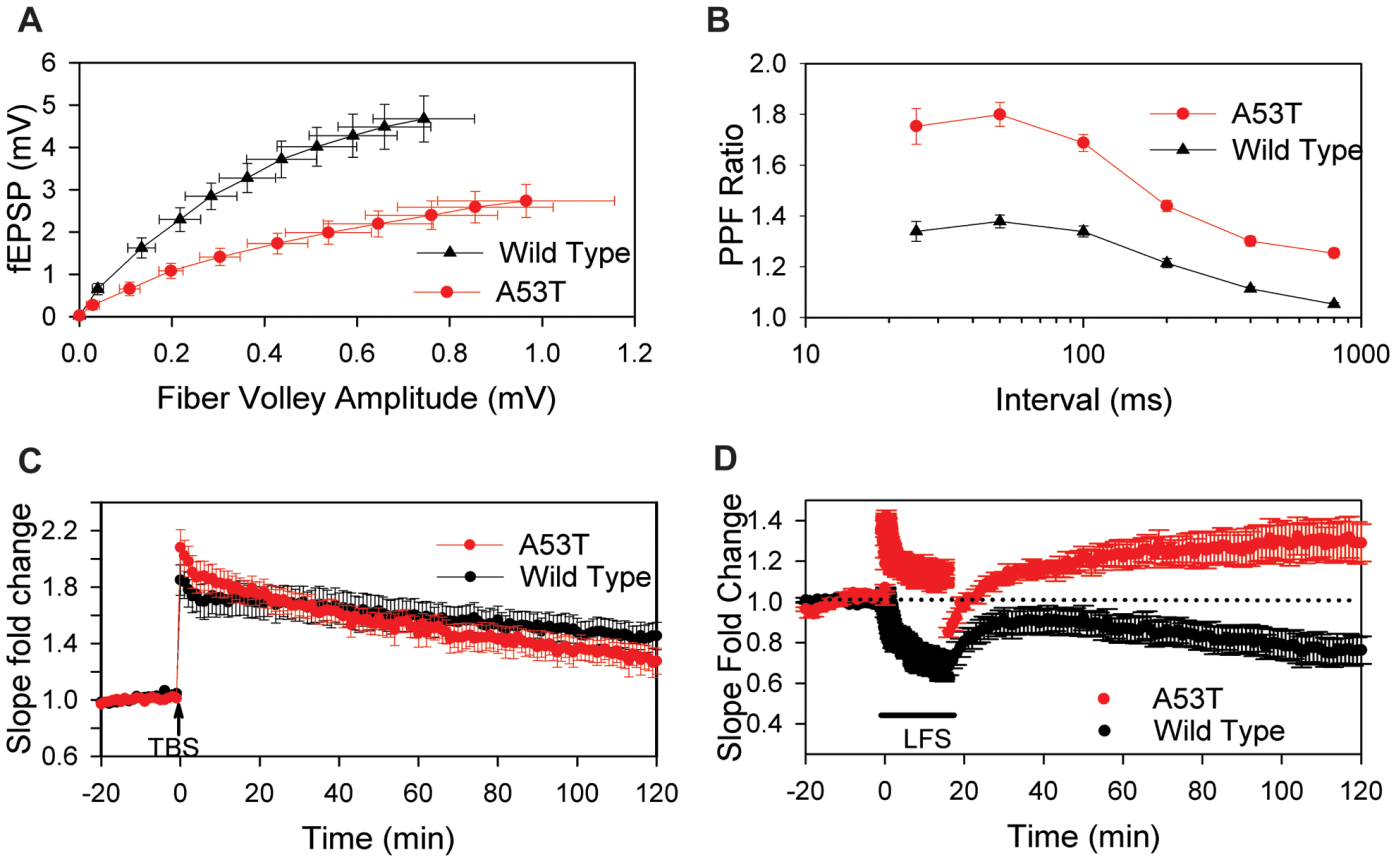
Synaptic deficits present in the CA1 region of A53T mice as early as 2 months. Field potentials were evoked by stimulating the Schaffer collaterals with a concentric bipolar electrode and recorded with glass electrodes in the stratum radiatum of CA1. Basal synaptic transmission is significantly impaired in 2 month A53T mice compared to wild type littermates (A). Paired-pulse facilitation (PPF) is significantly enhanced in 2 month-old A53T mice (B). Theta burst stimulation (TBS) induces LTP similarly in 2 month-old wild type and A53T mice (C). Low frequency stimulation (LFS, 900 pulses at 1 Hz) induces short and long term depression in wild type mice; however, it induces both short and long term potentiation in A53T mice (D). Data plotted as Mean+/− SEM. fEPSP = Field Excitatory Post-Synaptic Potentials, mV = millivolts, min = minutes.

In order to identify a potential mechanism related to the early onset behavioral deficits described above, we opted to examine synaptic function in young A53T mice. To determine whether expression of mutant A53T human α-syn alters basal synaptic transmission in the CA1 region of the hippocampus, the peak amplitude of fEPSPs was measured in slices prepared from 2-month old mice at various stimulation intensities then normalized to their corresponding fiber volley amplitude to generate the input-output relationship (I-O curve). As seen in Figure 7A, basal synaptic transmission in mice homozygous for A53T was greatly reduced compared to wild-type littermates. Next, we examined paired pulse facilitation (PPF), a measure of short-term plasticity, in these mice. We found that PPF was enhanced in A53T mice relative to wild-type littermates and this difference is maintained even at relatively long (1s) intervals (paired pulse ratio: 1.51±0.03 (WT) vs. 1.71±0.04 (A53T) at 50 ms and 1.11±0.01 (WT) vs. 1.23±0.01 (A53T) at 800 ms (n = 11; 14, p<0.01); Fig. 7B). High frequency stimulation (triple theta burst stimulation, TBS) induced long-term potentiation (LTP) in a similar manner in both wild-type and A53T mice. There was no statistical difference between the two groups 5 minutes after stimulation (fEPSP slope fold change 2 hours post-induction: 1.65±0.06 (WT, n = 12) vs. 1.59±0.06 (A53T, n = 14, p>0.05); Fig. 7C); however, within the first 2 minutes after TBS, slices from A53T mice showed a significant enhancement in potentiation compared to wild-types. This effect might be expected due to the enhanced paired pulse facilitation seen in the A53T mice. Low frequency stimulation (900 pulses at 1 Hz), a protocol designed to induce long term depression in the CA1 region, produced a modest decrease of fEPSPs in wild-type mice as expected. However, in slices from A53T expressing mice, low frequency stimulation produced a significant potentiation of fEPSPs in A53T, which was stable throughout the time of recording (fEPSP slope fold change 2 hours post-induction: 0.76±0.07 (WT n = 7) and 1.29±0.09 (A53T n = 7), p<0.001; Fig. 7D). All experiments (except for LTD) were also conducted in slices prepared from 9-month-old wild type and A53T mice with similar results (Fig. S5).

## Discussion

The human A53T mouse model studied here has been previously characterized in the context of α-syn accumulation and aggregation [13], [14], [23], [32]; however, the course of age-related phenotypic deficits has not been characterized in detail. This type of characterization is important for identifying sensitive and reliable outcome measures in preclinical models in order to facilitate the development of pharmacological therapies pertinent to PD [38]. Therefore, in the present studies we performed a battery of motor, sensorimotor, cognitive and physiological tests to assess the impact of α-syn accumulation and deposition on normal function prior to the onset of motor disability (ie., paralysis). In line with previous work, these mice develop age-related α-syn pathology and gross motor dysfunction corresponding to the onset of disease (8–17 months). In addition, we are the first to report an age-related disruption in spatial memory in this model. Furthermore, it is notable that these mice exhibit profound fine/sensorimotor deficits and synaptic dysfunction long before α-syn accumulation/aggregation or obvious motor symptoms are evident. This finding is especially important because PD patients often present with sensorimotor and non-motor symptoms long before the onset of motor dysfunction [39], [40], [41], [42]. Although the exact mechanisms responsible for eliciting these impairments are unknown, our findings suggest this model may be useful for examining the underlying cause of pre-motor symptoms in PD patients. Additionally, this model could be used for examining disease-modifying therapeutics as it provides specific endpoints for examining both early and late symptoms related to PD.

Our initial studies verified the expression of human α-syn in the A53T mouse brain. We show human-specific α-syn is ubiquitously expressed in both neurons and neuropil of homozygous animals (Fig. 1A). Previous studies have shown human α-syn was expressed in brains of 9 month old mice [14] and triton insoluble α-syn accumulated in the spinal cord and cerebellum of aged (11–12 months) animals [25]. Here, we report human-specific, soluble α-syn expression in brain increases significantly between 2 and 6 months of age, and remains constant at 12 months. We also observed α-syn aggregates dispersed throughout the cortex, hippocampus, brain stem and cerebellum in 6 and 12 month old mice. A few aggregates were seen in the 2-month animals, but they were barely visible and extremely rare. We also examined the onset of disease (i.e. motor disability) in these mice and found that as a group they became symptomatic between 8–17 months. Visual inspection of homozygous mice revealed no obvious gross motor dysfunction (ataxia or paralysis) until the age of ∼8 months when animals began to develop a severe motor phenotype depicted by decreased grooming, weight loss and reduced ambulation. Eventually, mice succumb to freezing bouts, ataxia and limb paralysis at which time they were euthanized. Combined, these findings demonstrate the A53T mouse exhibits vast expression of human α-syn throughout the brain that increases and aggregates with age and this corresponds to the onset of disease, as defined by motor disability. However, it is important to note that the window of time required for 100% of mice to become symptomatic was 9 months, which is a major limitation when using this animal model. It becomes problematic when examining a finite endpoint for pathological or behavioral changes since only a percentage of mice will be exhibiting symptoms at a given time. This variability may be overcome by significantly increasing group size or using disease onset (ie., paralysis) as an endpoint.

Tests of locomotor activity (LMA), rotarod and gait analysis are frequently utilized to evaluate gross motor function and coordination in neurodegenerative mouse models [43], [44], [45], [46]; therefore, we evaluated 2, 6 and 12 month A53T mice in these tests to establish the onset and extent of gross motor impairment. Results from spontaneous locomotor testing show the A53T mice display a hyperactive phenotype at 6 and 12 months. This finding is consistent with previous studies showing that enhanced locomotor activity is associated with mutant α-syn [23], [32]. Additionally, we report the A53T mice exhibit shortened stride length and speed at 12 months of age. Importantly, this finding relates to the human disease as altered gait is a symptom of PD [47]; specifically, patients’ have a shortened stride length, which results in a shuffling gait [48]. In contrast, we found that A53T mice were not initially impaired on the rotarod task, but instead exhibited a significant enhancement in motor coordination that suddenly decreases at 12 months of age. Data from individual trials highlight the separation between wild type and homozygous mice at 2 and 6 months; then, at 12 months there is a substantial decrease in latency for both genotypes. This significant reduction in latency exhibited by 12-month-old wild type mice was unexpected as they do not exhibit motor abnormalities; however, rotarod performance has been shown to be affected by differing body weights [49]. Enhanced latency is often exhibited by mice that weigh less, while reduced latency is seen in heavier mice since they have difficulty staying on the rod [50]. Therefore, the sudden decrease in latency for 12-month-old wild type mice is likely due to their significant weight gain. Regardless of the potential confound of body weight, 12-month-old A53T mice demonstrated a significant deficit in their ability to maintain balance on the rotarod relative to 2 and 6 month old A53T mice, most likely due to the development of motor perturbations at this age (i.e., hyperactivity and shortened gait). In summary, A53T mice develop motor abnormalities with age; however, this phenotype does not indicate a gradual or progressive impairment in motor ability (like the human disease), but more likely represents an abrupt shift or change that may be related to increased α-syn levels or aggregates.

It is interesting that a PD model over-expressing mutant α-syn exhibits an increase in activity with age instead of a reduction; however, previous studies show mice expressing the human A53T mutation do not experience overt loss of dopamine (DA) neurons, but instead exhibit alterations in DA signaling and striatal plasticity [32], [51], [52]. One study in particular [32] demonstrated that this age-related increase in locomotor activity is specific to the A53T form of α-syn as mice over-expressing the A30P mutation did not elicit the same phenotype. The authors claim the hyperactivity results from a decrease in DA transporter (DAT) expression in the striatum and nucleus accumbens, which is associated with reduced striatal DA uptake. Furthermore, D1 receptor expression is elevated within the SN causing increased sensitivity to DA. These findings have been replicated by several other groups [23], [32], [53], [54] and suggest the A53T mice may represent a model of early dopaminergic dysfunction.

Although the etiological basis of anxiety in PD is likely multifactorial, DA dysfunction relating to DAT expression has been implicated in its pathogenesis [55], [56]. This is important as anxiety is a co-morbid disorder that presents with PD [57], [58], often before the onset of motor symptoms [57]. In line with previous studies [20], [23], we found that 12 month A53T mice spent significantly more time in the center of an open field and exhibited a modest trend for decreased SIH compared to wild type littermates, suggesting these animals develop a hypo-anxious phenotype with age. While SIH is not confounded by motor alterations, the center time data should be interpreted with caution as these data correspond to the age-dependent increase in locomotor activity, thus making it difficult to ascertain whether mice are truly less anxious or if they spend more time in the center field due to hyperactivity. However, attenuation of the acoustic startle response may be indicative of an anxiolytic-like effect as startle is exacerbated with anxiogenics [59], [60] and attenuated with anxiolytics [61], [62], thus suggesting A53T mice develop an anxiolytic phenotype with age. Although this finding is contrary to the increased anxiety phenotype that often presents in human PD, it should be noted that mutations in α-syn are only one of several mechanisms implicated in the disease. As such, our findings do not exclude the involvement of other pathways, which may contribute the co-morbid anxiety experienced by PD patients. However, the robust changes in anxiety exhibited by this model suggest it may still prove useful for investigating how the A53T mutation affects the limbic system and alters normal affect (independent of DA neuron loss).

The characteristic clinical symptoms of PD are frequently accompanied by impairments in cognitive function. Approximately 20% of PD patients develop dementia [63]; however, mild cognitive impairment [64] is also a well recognized feature of the disease and is an important predictor for quality of life [65], [66]. To this end, we sought to determine whether A53T mice display cognitive impairments. The Y-maze procedure utilized here tests hippocampal-dependent short-term spatial memory and is based on the innate preference of mice to remember and explore an arm that has not been previously explored. Our results show homozygous mice at 2 months of age display normal novelty recognition; however, at 6 and 12 months, mice display a significant deficit, suggesting a disruption in short term memory. The exact role of α-syn in cognition is unclear; however, a role for DA in working memory has been shown in both patients and healthy subjects [67], [68]. Additionally, there is increasing evidence that DA plays a role in episodic memory [69]. In light of these findings, the age-dependent spatial impairment evident in the A53T model may be the result of a dysfunctional DA system (although additional testing should be done to confirm this hypothesis). In summary, we find A53T mice develop changes in motor ability, anxiety and cognition with age and while the exact mechanisms involved in these behavioral alterations are unknown, it is most likely due to perturbations in the DA system that may or may not be associated with mutant α-syn [23], [70]. Furthermore, these late-onset deficits correspond to late-onset symptoms in PD patients suggesting the A53T mouse may have some translational value when it comes to understanding the role of α-syn relative to DA function in PD.

While the cardinal features of PD include various perturbations in motor ability (i.e., bradykinesia, postural instability, tremor, etc.), evidence suggests the pre-motor symptoms have a more profound effect on quality of life are less well tolerated by patients [40], [71]. To this end, we evaluated the temporal onset of select fine- and sensorimotor behaviors in A53T mice to determine whether these behaviors correspond to α-syn pathology. We were able to detect significant impairments in homozygous mice as early as 1–2 months of age in all tests administered. We found A53T mice groom significantly less than their WT littermates and young A53T mice are slower and less proficient than their wild-type littermates at nest building. Combined, these behaviors suggest A53T mice have issues with fine motor and/or orofacial muscle control making it difficult for them to shred cotton and groom themselves. This finding is highly relevant from a translational aspect as mastication and orofacial function are impaired in patients with moderate to advanced PD [72], [73], [74]. Alternatively, the DA system is linked to motivation and attention [75], suggesting the deficits in grooming and nesting may not be the result of altered motor ability, but instead, due to a lack of motivation. To test this hypothesis, we opted to evaluate performance in a task that measures an involuntary response, the acoustic startle reflex. This behavioral paradigm is readily translated across species and is clinically relevant as the auditory startle reaction has been shown to be impaired in parkinsonian disorders [76]. Furthermore, this test has been utilized as an early diagnostic for various neurodegenerative disorders including PD [76], [77]. Consistent with findings in PD patients, we found that A53T mice exhibit significant reductions in the startle response at an early age, long before the onset of motor disability. Alternatively, the attenuated acoustic startle response may also be an indicator of an anxiolytic-like phenotype, which would be consistent with our results in other anxiety measures as discussed above. However, there was a concern that these mice may exhibit hearing loss due to transgene expression during neural development [78], [79], which would confound findings. Therefore, to ensure the startle reflex deficits were due to an atypical brainstem reflex response and not due to hearing loss, we confirmed normal hearing ability in the A53T mice via the auditory brainstem response. Taken together, our findings show the A53T mice, like PD patients, exhibit profound fine motor, sensorimotor and gating deficits prior to the onset of gross motor disability.

Previous studies examining synaptic function in the striatum of A53T mice have shown a decrease in LTD induced by high frequency stimulation [53], [54]. This deficit in synaptic plasticity was age-dependent and was only evident in older mice (>18 months of age) [53], [54]. Additionally, acute DA application and L-3,4-dihydroxyphenylalanine (L-DOPA) treatment could not attenuate striatal synaptic deficits in aged A53T mice [54], which suggests these deficits are not due to a loss of dopamine, but rather altered DA transmission. Here we examined synaptic function in the CA1 region of the hippocampus where we found reduced basal synaptic transmission and enhanced paired pulse facilitation in A53T mice. In contrast to the aforementioned studies, these deficits were present at the earliest time point examined (1–2 months) and subsequent studies found this effect was independent of age, as 9 month A53T mice also displayed these alterations. Furthermore, hippocampal LTP was unaffected in A53T mice. In contrast, hippocampal LTD was dramatically altered at 2 months and the low frequency stimulation, which normally produces a modest depression, produced potentiation of the fEPSP. These findings demonstrate that A53T over-expression is associated with synaptic dysfunction in the absence of neurodegeneration and before the mice exhibit gross motor symptoms. However, previous studies in both *in vitro* and *in vivo* systems demonstrate impaired synaptic transmission due to the over-expression of wild type α-syn [80], [81], which is analogous to the inhibition of basal synaptic transmission reported here. Therefore, the altered synaptic function observed here may not be specific to the A53T mutation, but rather to the over-expression of α-syn; thus, identification of synaptic effects specific to the A53T mutation would require further study. Taken together, the significant changes in basal synaptic transmission that occur in young mice prior to the onset of behavioral Parkinsonian-like symptoms indicate these mice may be useful in understanding mechanisms involved in the early stages of PD.

Historically, these mice have been utilized to examine the effect of α-syn point mutations on normal function [13], [14], [23] as well as to evaluate therapies aimed at reducing α-syn [82]; however, without tangible behavioral endpoints, it is difficult to ascertain the role of α-syn or the effect of its clearance on functional outcomes. This study demonstrates the phenotypic deficits in A53T mice are established in an age-dependant manner and can be temporally clustered into early deficits observed in 1–2 month old mice and late deficits in mice >6 months. While these changes essentially parallel early- and late-onset symptoms in PD patients, caution must be taken when interpreting these findings. Although these mice develop pathological changes that correspond to behavioral manifestations, the behavioral changes do not seem to progress with time, but rather develop in an all or none fashion. Perhaps examination of an additional time point between 2 and 6 months would reveal a more gradual progression; however, the current results show that mice either exhibit deficits early on (probably from birth) or develop them by 6–12 months. Since the late stage manifestations correspond with an increasing accumulation of soluble and aggregated α-syn, it suggests the late-onset phenotype may be dependent on α-syn pathology and could therefore be attenuated by clearing the mutant protein. In fact, we utilized this model to evaluate the effects of an autophagy enhancer on disease progression (manuscript in review). However, a major limitation of these mice is that they do not develop late-onset deficits uniformly; in fact, the window of time required for these mice to become symptomatic introduces excessive variability, thereby making it difficult to assess effects of α-syn clearance on late-stage functional outcomes. Nevertheless, changes in α-syn pathology can be systematically evaluated with multiple repeats of large cohorts of aged animals (>12 months) with relative consistency. Alternatively, the early-onset symptoms appear in all mice by 1–2 months of age (perhaps even earlier), which provides a more reliable endpoint (in these mice) for evaluating therapeutic strategies in the context of early PD. Interestingly, these early deficits do not correlate with α-syn accumulation or aggregation, suggesting additional mechanisms may underlie the early symptoms of PD. Alternatively, it may indicate that cell types or brain regions linked to the earlier symptoms are more susceptible to α-syn alterations and/or toxicity and do not compensate to the same degree as the motor system (which is impacted late in the course of disease). Overall, this study illustrates the temporal progression of deficits in the A53T model, which recapitulates many of the key features of PD, including α-syn neuropathology, derangements in cognition and synaptic transmission, and motor deficits. Although this model does not appear to develop a progressive motor phenotype or overt neurodegeneration as seen in human PD, it remains a useful tool with tangible biochemical and behavioral endpoints for testing therapeutics relevant to human disease. Furthermore, the evidence of prodromal symptoms occurring independent of α-syn pathology and/or neurodegeneration highlights the importance of early intervention in PD.

## Supporting Information

Figure S1 A53T mice develop significant alterations in motor ability with age. A53T homozygote mice develop gross motor dysfunction with age. Graphs show time-course (over 60 minutes) for total distance travelled (A), horizontal activity (B) and vertical activity (C) in A53T homozygous mice at 2, 6 and 12 months (analyses depict data combined from two separate cohorts of homozygous mice; n = 20–25 per group). Digigait analyses depicting the stride frequency (speed) and length of both the left and right hind limbs for 2, 6 and 12 month A53T mice compared to wild type littermates (D). Graphs show rotarod time course for latency to fall over 5 independent trials for each age group (2, 6 and 12 months; E). Body weight is significantly increased in 6 and 12 month wild type mice compared to A53T mice (F). Unless otherwise noted, graphs are representative of data collected from 2–3 independent cohorts of animals with 8–15 mice per group. Data plotted as Mean+/− SEM. *p<0.05; s = seconds, cm = centimeters, M = month (TIF)

Figure S2 A53T mice do not exhibit alterations in locomotor indices during spatial memory testing. A53T and wild type mice perform similarly in various motor indices during both trials (T1 & T2) of the Y-maze test. There are no significant differences in the total distance traveled (A, B), the speed of travel (C, D), number of entries into arms (E, F) or the total time spent in arms (G, H), suggesting deficits in novelty recognition (i.e., spatial memory) exhibited by 6 and 12 month A53T mice is not due to motor alterations. Data plotted as Mean+/− SEM. N = 11–15 mice per group. T1 = trial 1, T2 = trial 2, s = seconds, WT = wild type, cm = centimeters (TIF)

Figure S3 Nestlet Rating Scale. The ability of both homozygous and wild type mice to build nests was assessed by assigning a score (0–6) based on the quality of nests built after 1, 3, 5 and 24 hours. At each time point, a blinded investigator administers a score based on how much of the nestlet is shredded, whether the shredded pieces are gathered into a nest or spread throughout the cage and how compact the nest is. A score of 0 was given if a nestlet was untouched (A). A score of 1 was given if a nestlet had been shredded up to 10% (B). A score of 2 was given if the nestlet was shredded up to 25% and scattered (C). A score of 3 was given if up to 50% of the nestlet had been shredded and somewhat gathered (D). A score of 4 was given when up to 75% of nestlet was shredded, pieces are gathered and connected (E). A score of 5 was given when 75–100% of nestlet is shredded into a localized and defined nest with walls (F). A score of 6 was given when 100% of nestlet is shredded and built into a tight 3D nest that rises above the mouse and is localized in one corner (G). Additionally, 0.5 points was given when all of the criteria was met for the lower whole number and some, but not all, of the criteria were met for the next number on the scale. (TIF)

Figure S4 A53T and wild type mice exhibit divergent responses to acoustic stimulus with age. At all ages, startle amplitude is substantially lower in A53T mice (<15 mN) compared to their wild type littermates (<45 mN) (A, B). As the auditory stimulus increases (>110 dB), wild type and A53T mice display divergent startle responses depending on age. At the 120 dB stimulus, the startle response is significantly decreased at 6 and 12 months compared to the 2 month group in wild type mice (A); however, the startle response is increased at 6 and 12 months in the homozygous A53T mice (B). Graphs are representative of data collected from 2 independent cohorts of animals with 8–15 mice per group. Bonferonni post hoc test *p<0.05; **p<0.001; ***p<0.0001; mN = millinewtons, dB = decibels, WT = Wild type (TIF)

Figure S5 Synaptic deficits evident in 9-month-old A53T mice. Nine month old A53T mice have altered CA1 basal synaptic transmission compared to wild type littermates (A). Paired-pulse facilitation (PPF) is significantly enhanced in 9 month old A53T mice (B). Theta burst stimulation (TBS) induces LTP similarly in 9 month old wild type and A53T mice (C). Data plotted as Mean+/− SEM. fEPSP = Field Excitatory Post-Synaptic Potentials, mV = millivolts, min = minutes (TIF)

Table S1 Individual grooming behaviors in 2, 6 and 12 month wild type (WT) and A53T homozygous (HO) mice. Mice develop deficits in grooming behavior with age. No significant differences exist between WT and HO mice at 2 months of age; however, impairments become evident in the 6 and 12 month groups. (DOCX)
