# Supplementary figures and images for: Behavioral Characterization of A53T Mice Reveals Early and Late Stage Deficits Related to Parkinson’s Disease
Source: PLoS One. 2013 Aug 1;8(8):e70274. doi: 10.1371/journal.pone.0070274 (PMC3731353; doi:10.1371/journal.pone.0070274)

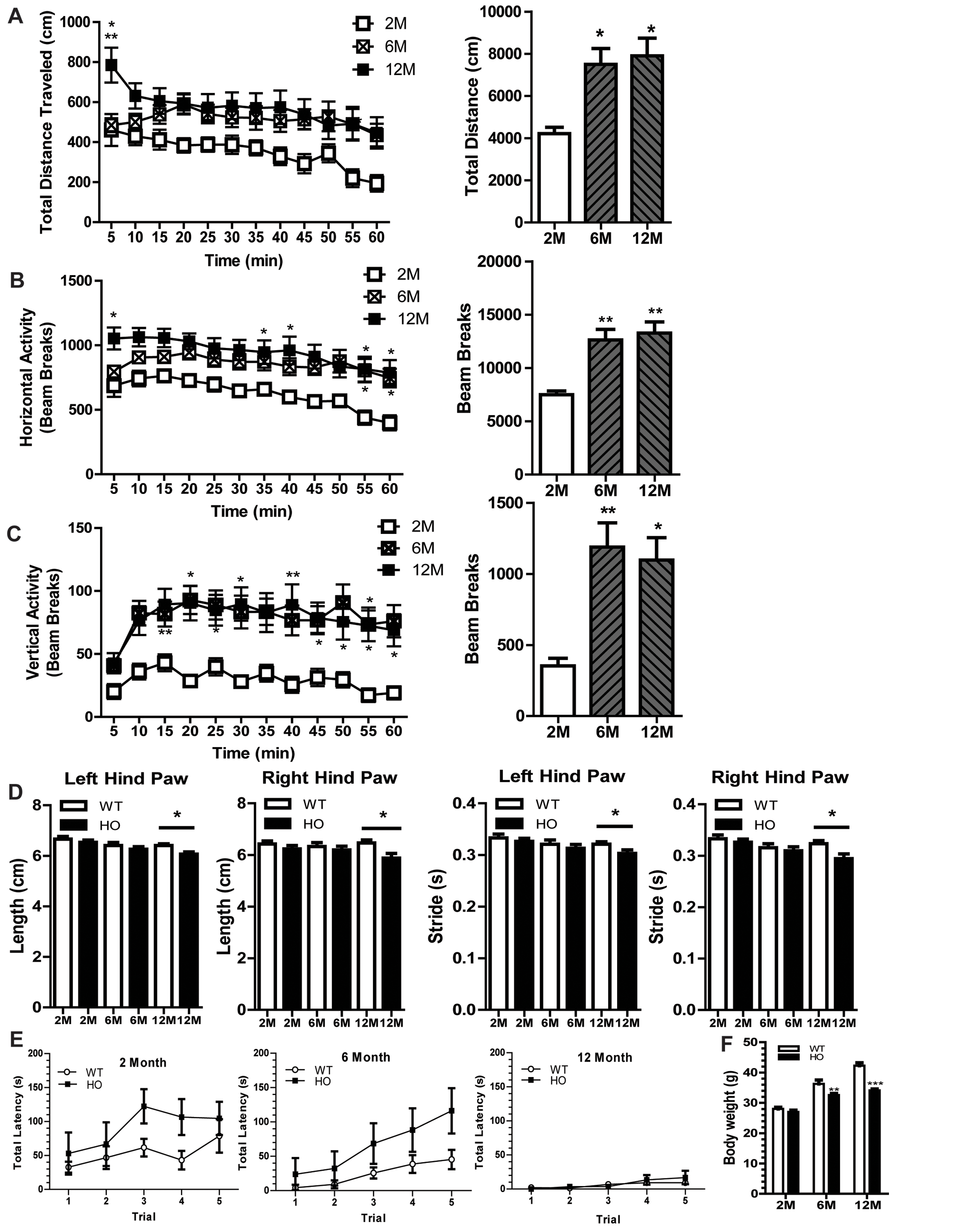

Supplement: Figure S1 — (TIF) [file pone.0070274.s001.tif]

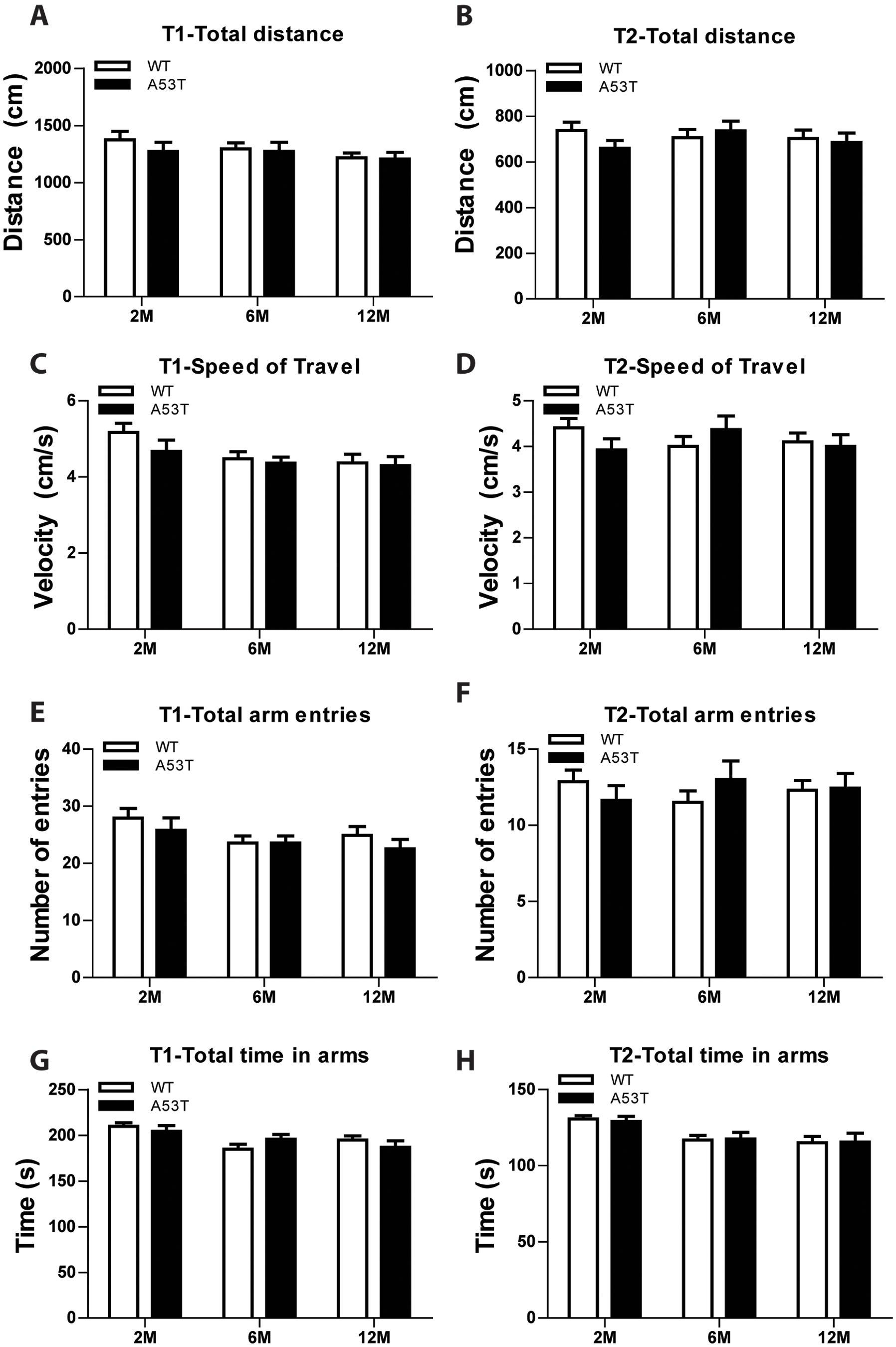

Supplement: Figure S2 — (TIF) [file pone.0070274.s002.tif]

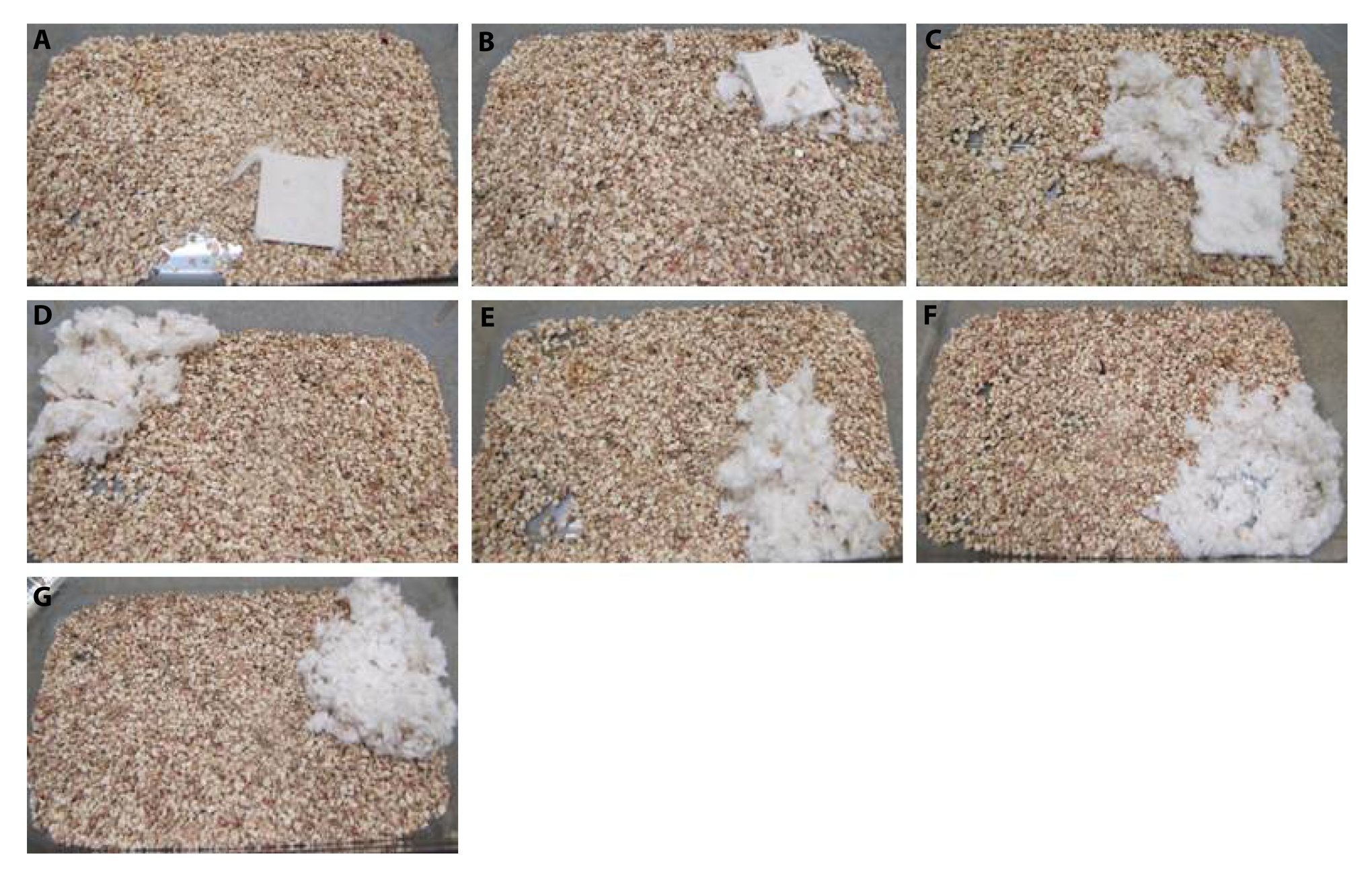

Supplement: Figure S3 — (TIF) [file pone.0070274.s003.tif]

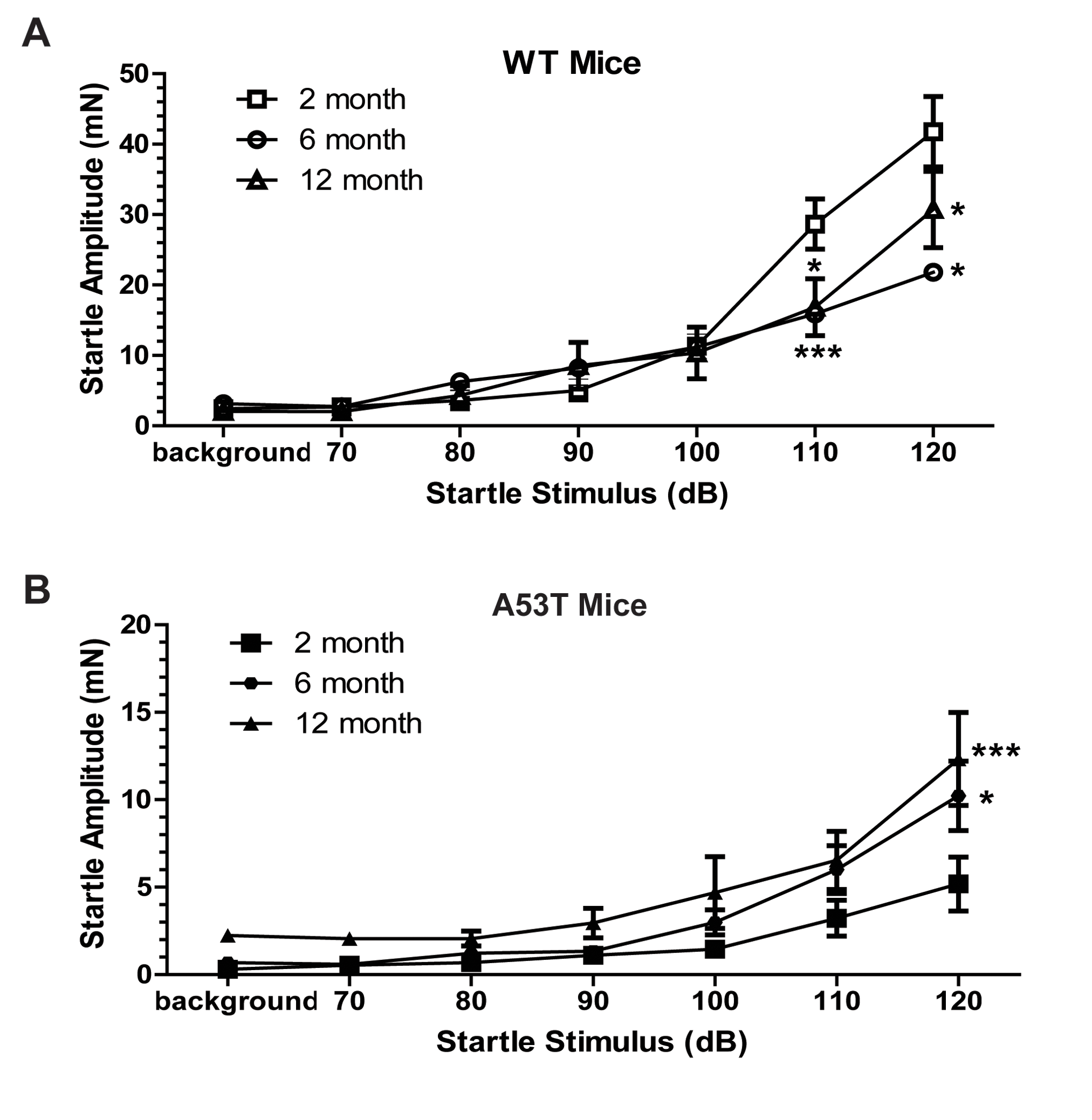

Supplement: Figure S4 — (TIF) [file pone.0070274.s004.tif]

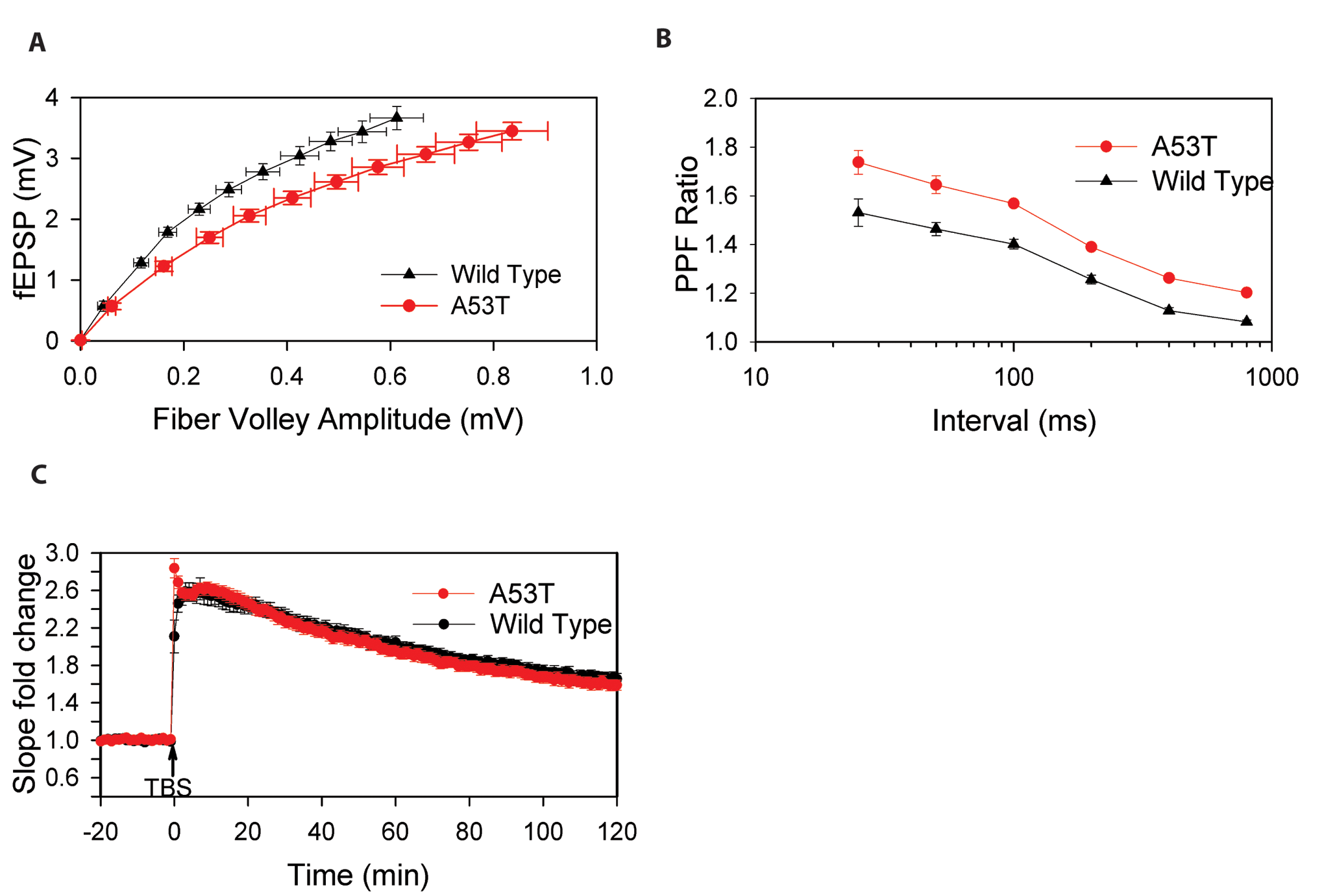

Supplement: Figure S5 — (TIF) [file pone.0070274.s005.tif]
