# Supplementary material for: Behavioral Characterization of A53T Mice Reveals Early and Late Stage Deficits Related to Parkinson’s Disease
Source: PLoS One. 2013 Aug 1;8(8):e70274. doi: 10.1371/journal.pone.0070274 (PMC3731353; doi:10.1371/journal.pone.0070274)
Supplement: Table S1 — (DOCX) [file pone.0070274.s006.docx]

Supplemental Table 1. Individual grooming behaviors in 2, 6 and 12 month wild type (WT) and A53T homozygous (HO) mice. Mice develop deficits in grooming behavior with age. No significant differences exist between WT and HO mice at 2 months of age; however, impairments are evident in the 6 and 12 month groups.

| **Mean + SEM** | | | | | | | | | |
| --- | --- | --- | --- | --- | --- | --- | --- | --- | --- |
|  | **Two Month** | | | **Six Month** | | | **Twelve Month** | | |
| **Grooming Behavior** | **WT** | **HO** | **P value** | **WT** | **HO** | **P value** | **WT** | **HO** | **P value** |
| **brush** | 0.2727 ± 0.1408 | 0.09091 ± 0.09091 | p>0.05 | 0.2500 ± 0.1637 | 0.2857 ± 0.1844 | p>0.05 | 1.750 ± 0.8183 | 0.2857 ± 0.1844 | p>0.05 |
| **clean** | 2.545 ± 1.498 | 2.455 ± 0.9569 | p>0.05 | 12.38 ± 3.817 | 3.571 ± 0.8411 | **p=0.0549** | 25.13 ± 7.128 | 15.57 ± 10.14 | p>0.05 |
| **clean limb** | 8.818 ± 1.985 | 3.909 ± 2.060 | p>0.05 | 11.38 ± 2.456 | 2.143 ± 0.5533 | **p<0.01** | 16.00 ± 2.428 | 2.429 ± 0.4286 | **p<0.01** |
| **comb** | 1.273 ± 0.5062 | 1.091 ± 0.4946 | p>0.05 | 4.250 ± 1.206 | 0.8571 ± 0.4041 | **p<0.05** | 6.875 ± 2.048 | 3.143 ± 1.844 | p>0.05 |
| **groom** | 7.273 ± 2.838 | 2.182 ± 1.127 | p>0.05 | 10.25 ± 2.297 N=8 | 5.000 ± 2.507 N=7 | p>0.05 | 21.25 ± 3.178 N=8 | 8.000 ± 3.471 N=7 | **p<0.05** |
| **groom limb** | 71.91 ± 7.719 | 67.18 ± 7.846 | p>0.05 | 136.6 ± 9.504 | 100.7 ± 11.37 | **p<0.05** | 190.4 ± 18.52 | 183.6 ± 58.05 | p>0.05 |
| **scratch** | 0 | 0 | p>0.05 | 0.375 ± 0.1830 | 0 | -- | 0.5000 ± 0.2673 | 0.2857 ± 0.2857 | p>0.05 |
| **sniff** | 176.5 ± 25.16 | 123.1 ± 18.20 | p>0.05 | 197.0 ± 38.17 | 109.4 ± 12.25 | **p=0.0603** | 206.1 ± 35.71 | 112.0 ± 19.92 | **p<0.05** |
| **wash** | 1.182 ± 0.5191 | 0.4545 ± 0.2073 | p>0.05 | 2.750 ± 0.9590 | 0.4286 ± 0.4286 | **p=0.0556** | 6.000 ± 1.254 | 2.857 ± 1.370 | p>0.05 |
| **wipe** | 1.273 ± 0.6338 | 0.1818 ± 0.1220 | p>0.05 | 3.000 ± 0.8452 | 0.4286 ± 0.2020 | **p<0.05** | 12.88 ± 2.333 | 0.5714 ± 0.5714 | **p<0.001** |
| **wipe limb** | 13.73 ± 4.032 | 6.182 ± 2.044 | p>0.05 | 40.38 ± 5.788 | 6.000 ± 1.291 | **p<0.001** | 68.63 ± 7.878 | 13.86 ± 4.574 | **p<0.001** |

*Groom = subtle movements of the paw, usually on the face; Wipe = brushing of the fur by the paw; Clean = vigorous cleaning by the paw; Scratch = vigorous and rapid movement of the foot; Sniff = subtle movements of the head; Groom = small movements of the head, usually includes arms; Comb= small, short movements of the head and arms, usually on body fur; Wipe = small, but long movements of the head; Brush = large, short movements of the head; Clean = large, long movements of the head; Wash = vigorous movements of the head, usually looks like deep grooming (biting motion)*
